# Process characterisation of continuous ring layer wet granulation at small scale

**DOI:** 10.1016/j.ijpx.2025.100454

**Published:** 2025-11-23

**Authors:** Lukas Bahlmann, Jan Henrik Finke, Arno Kwade

**Affiliations:** a Institute for Particle Technology (iPAT), Technische Universität Braunschweig, Volkmaroder Straße 5, 38104 Braunschweig, Germany; b Center of Pharmaceutical Engineering (PVZ), Technische Universität Braunschweig, Franz-Liszt-Straße 35A, 38106 Braunschweig, Germany

**Keywords:** Continuous manufacturing, Ring layer, Wet granulation, Microstructure, Process parameters

## Abstract

Ring layer granulation is a wet granulation process, which can be applied for the continuous production of pharmaceutical granules as an alternative to other continuous granulation techniques like twin screw granulation or continuous fluidised bed granulation. However, the ring layer process itself has so far been the subject of only little fundamental scientific investigation. Additionally, for the few published studies, medium to large scale ring layer granulators were utilized for which large quantities of sample material was necessary. This shortfall is addressed in the present study, in which the unique lab scale ring layer granulator Granucon®1, giving the possibility of small scale experiments and production campaigns, was investigated. The ring layer process was studied for the wet granulation of microcrystalline cellulose, an insoluble primary material, with variation of the process parameters tip speed, binder supply rate and solid feed rate. Moisture content showed the most significant effect on the granulation results, while shaft speed and solid feed rate influence the residence time of the granules inside the ring layer granulator. Further, while a certain shaft speed had to be reached for the ring layer to form, it also had a strong effect on the granule morphology due to its effect on the mechanical stress acting on the granules.

## Highlights

• Ring layer granulation is investigated on a unique small lab scale plant.
• Short residence times are achieved, which are dependent on tool tip speed and solid feed rate in particular.
• Formation of the ring layer is dependent on the tool tip speed and crucial for sufficient granulation results.
• Liquid-to-solid ratio shows the most pronounced effect on final granule size.
• Tool tip speed influences the shape of the granule size distribution.
• Increase of mechnical stress in the process increases granule density and sphericity, but decreases final granule size.

## Graphical abstract

**Figure:**
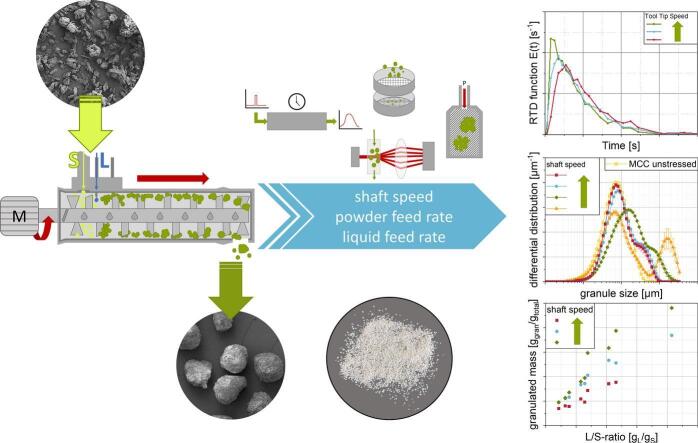

## 1 Introduction

Granulation is a fundamental technology in process industries for products such as food, chemicals and pharmaceuticals. In the pharmaceutical industry, granulation processes play a particularly important role in the production of solid dosage forms. By agglomerating individual powder particles into larger clusters, their properties are specifically modified to have a positive impact on the subsequent process chain. These include improved flowability, reduced segregation effects, reduced dust formation and improved tableting (Iveson et al., 2001). Wet granulation is often used, in which powder is brought into intensive contact with a binder liquid so that the primary particles arrange themselves into larger, solid agglomerates by means of liquid-solid bridges which turn to solid binder bridges after drying of the granules (Ennis et al., 1991; Iveson and Litster, 1998).

Continuous processes for wet granulation include twin screw granulation, fluidised bed granulation or ring layer granulation (Portier et al., 2021; Diez et al., 2018; Heinrich, 2002; Järvinen et al., 2015; Meng, 2018). Twin screw granulation, in particular, was investigated as a pharmaceutical granulation process as early as 1986 and has since been further developed in numerous studies (Gamlen and Eardley, 1986; Keleb et al., 2004; Kyttä et al., 2020; Forster et al., 2023) and integrated into the first process lines for fully continuous production, e. g. QbCon® by L.B. Bohle Maschinen+Verfahren GmbH, Germany and ConsiGma® by GEA Group AG, Germany. Twin screw granulation is a flexible process because of the many ways in which individual screw elements can be configured, but it is also susceptible to variations (Djuric and Kleinebudde, 2008; Kumar et al., 2016; Vercruysse et al., 2015).

Another method of continuous wet granulation is ring layer granulation (RLG). In ring layer granulation, similar to a continuous blender, the powder is fed into a horizontal process chamber. In there, the powder particles hit a high-speed rotating shaft equipped with different tools (pins and blades). The centrifugally accelerated particles form a dynamic, concentric layer on the inner wall of the cylindrical process chamber around the shaft. After binder liquid is introduced into the process chamber, the moist powder is agglomerated into granules under high shear rates in the narrow gap between the cylinder wall and the mixing tools and continuously discharged from the granulator (Meng et al., 2019; Meng et al., 2016). To date, there have been few scientific studies on ring layer granulation. These are mainly concerned with the comparison of this method with other discontinuous and continuous wet granulation processes (Järvinen et al., 2015; Meng, 2018; Meng et al., 2019; Meng et al., 2016; Meng et al., 2017; Kulju et al., 2016). For the ring layer process itself it was found, that with increasing shaft rotation speed and L/S-ratio, granule size and density can be significantly increased (Järvinen et al., 2015; Meng et al., 2016). In a study comparing the continuous RLG to high shear and fluidised bed granulation as batch processes, continuous ring layer granulation can produce equivalent granules in terms of pharmaceutical quality, with the RLG process being more easily controlled than the batch processes due to nearly linear responses of the granule size with the variation of shaft speed and binder flow rate (Järvinen et al., 2015). Compared with continuous twin screw granulation, ring layer granulation is also an adjustable process, despite the limited variability of the granulation tools. It has even been possible to achieve a lower variance in granule properties (Meng et al., 2016). The adaptability of the process results from the good controllability of the granulation process via the process parameters, with liquid saturation and speed being the most important ones (Meng et al., 2019; Meng et al., 2016).

This study used a unique small scale ring layer granulator for the continuous production of granules. This is the first time, a ring layer granulator of this scale is investigated. While the next larger scale version of this granulator used in the previously mentioned studies works with throughputs of 10 to 80 kg/h (Järvinen et al., 2015; Meng et al., 2016), the working range of this lab scale granulator is approximately between 2 and 10 kg/h, depending on the material used. This is particularly advantageous for research and development activities due to the low material consumption and it directly allows for scale-up free small scale production campaigns, e.g. for clinical studies or even market supply of orphan drug products. Consequently, the aim of this study was to comprehensively characterise the continuous process of ring layer granulation on this uniquely scaled granulator. Although the focus of this study is not on scaling of the device, comparisons with the literature on larger scale RLG are made, showing similiarities and differences. Additionally, comparisons were made with other batch and continuous wet granulation processes, generating detailed process understanding.

Therefore, the influence of parameter variations on the process itself and the granule properties were elucidated. The widely used excipient microcrystalline cellulose (MCC) was used as the insoluble primary material due its robust performance in other granulation processes, especially regarding overwetting (Portier et al., 2020; Chitu et al., 2011a). Contrary to the previous studies on RLG, MCC was the only powder component used here. Hence, effects from soluble carrier components on the granulation process were avoided.

## 2 Materials and methods

### 2.1 Materials

Microcrystalline cellulose (MCC, Parmcel102 [median particle size measured by laser diffraction $x_{50,3,LD}$ = 69 ± 1 μm], Gustav Parmentier GmbH, Frankfurt a.M., Germany) was used as the base material. Kollicoat®IR (kindly provided by BASF SE, Ludwigshafen, Germany), a macrogol poly(vinyl alcohol) grafted copolymer, acted as wet granulation binder and was applied as an aqueous solution with a concentration of 15 % (*w*/w). Brilliant blue FCF (Carl Roth GmbH + *Co*. KG, Karlsruhe, Germany) was used as a dyeing agent, added to the binder liquid in respective experiments.

### 2.2 Continuous ring layer granulation

**Fig. 1:**
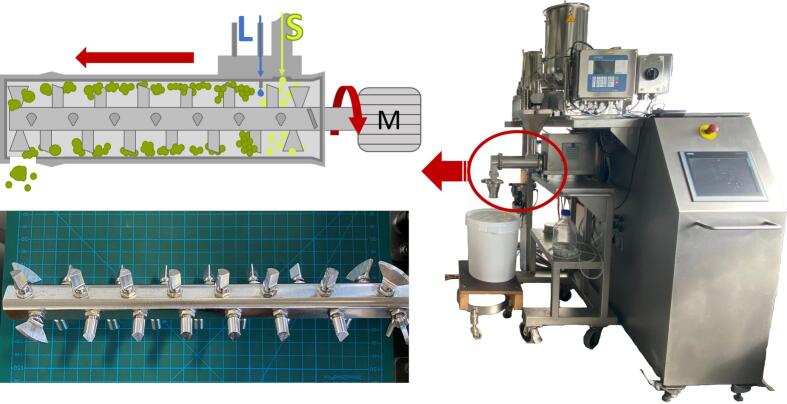
Image of the Granucon®1 lab scale ring layer wet granulation unit (right). In the upper left, a schematic figure of the ring layer process is displayed. In the lower left, a detailed image of the granulator shaft with the used tool configuration is shown.

The granulation was carried out using the lab scale ring layer granulator Granucon®1 (Gebr. Lödige Maschinenbau GmbH, Paderborn, Germany) (Fig. 1). As can be seen in Fig. 1, different tools are mounted at eight positions on each side of the four sides of the granulator shaft. While the blades at the beginning and the end of the shaft are mainly responsible for axial transport of the powder, the pins are responsible for shear transmission onto the wet powder and thus granule shaping. It is possible to change the position and alignment of the tools on the shaft as well as their distance to the inner wall of the granulation chamber. However, for the present study, the configuration was set as advised by the manufacturer. A detailed description of the tool configuration is given in the supplementary (Fig. S1). The diameter of the shaft equipped with the tools is 64 mm. The axial transport length through the granulator, from the powder inlet to the granule outlet, is 250 mm, while the granulation chambers diameter is 65 mm.

To evaluate the influence of the process parameters on the granule properties, the tool tip speed, powder feed rate and binder liquid addition rate were varied (Table. 1). The combination of the two latter parameters results in liquid-to-solid (L/S-) ratios between 0.043 g_L_/g_S_ and 0.420 g_L_/g_S_. Every combination of the parameter settings given in Table. 1 was investigated. A constant powder supply into the granulator was maintained by a twin screw feeder (KT20, Coperion K-Tron, Niederlenz, Switzerland). A gear pump (Masterflex® Ismatec, VWR International GmbH, Darmstadt, Germany) equipped with a suction shoe pump head (Micropump Inc., Vancouver, WA, USA) was used to add the binder solution. The binder addition rates are resulting from the pumps power settings (5 %, 10 % and 20 %).

**Table 1:** Settings of the individual process parameters in this study. Every combination of the three parameters was investigated.

Tool tip speed:	2.5 m/s, 4.9 m/s, 7.5 m/s, 9.9 m/s
Powder feed rate:	2 kg/h, 4 kg/h, 6 kg/h
Binder liquid addition rate:	4.3 g/min, 7.7 g/min, 13.8 g/min

For each run, there was a period of 1 min to fully enable the powder feed equilibration and the ring layer formation of the dry powder in the processing chamber. Afterwards, the binder supply was started. An equilibration time of at least 2 min was estimated to be sufficient for the wet granulation process to build up and reach steady state. Afterwards, 800 mL of wet granules were collected in a stainless steel pan and dried in a convective drying oven (Binder FED 400, Binder GmbH, Tuttlingen, Germany) for 24 h at 80 °C before further analysis. Additionally, reproducibility of the process was tested by carrying out three independent granulation runs at constant process parameters. Sufficient reproducibility was obtained with a coefficient of variation of the median granule size of 1.74 %, which is in a good range for between batch variability compared to other studies concerning continuous twin screw wet granulation (Keleb et al., 2004; Vercruysse et al., 2013). Further details can be found in the supplementary (Fig. S2, Table S2).

### 2.3 Granule characterisation

#### 2.3.1 Particle size measurements

The particle size distribution of the granules and the raw powder was determined by applying laser diffraction using the MasterSizer 3000 with an Aero S dry dispersion unit (both Malvern Panalytical Ltd., Malvern, UK). Prior to the particle size measurements, powders were sieved to remove granules larger than 2.5 mm. For each powder, three separate samples (*n* = 3) with a sample mass of approximately 5 g were measured and median as well as mean values ($x_{50,3,LD}\;$ and ${\bar{x}}_{1,3,LD}$, respectively) were calculated. The volume weighted mean of the particle size distribution ${\bar{x}}_{1,3,LD}$ was determined by:

(1) $${\bar{x}}_{1,3,LD}=\int_{x_{min}}^{x_{max}}x_{i}\cdot q_{3}\left(x_{i}\right)dx$$

with *x*_*i*_ being upper limit of the size intervall *i*, *q*_*3*_*(x*_*i*_*)* being the respective value of the density distribution function and *x*_*min*_ and *x*_*max*_ the lower and upper limit of the measured particle size range, respectively.

Additionally, sieve analysis (Retsch AS200 control, Retsch GmbH, Haan, Germany) of the starting material and the granules was carried out using the mesh sizes 1000 μm, 500 μm, 250 μm, 180 μm and 63 μm. After sieving, the mass fraction of the obtained granule fractions was calculated.

Further, since particle comminution in certain limits was observed, a value for the degree of particle size reduction (PSR) was calculated by using the median particle sizes of the unstressed primary material and the primary material stressed in the granulator at different tip speeds and solid feed rates:

(2) $$PSR=\frac{x_{50,3,LD,unstressed}-x_{50,3,LD,stressed}}{x_{50,3,LD,unstressed}}$$

where *unstressed* refers to particle sizes before passage through the RLG and *stressed* refers to particle sizes after the passage of the RLG under given process parameters.

#### 2.3.2 Granule morphology

The shape of the granules was assessed through dynamic image analysis (QICPIC with VIBRI dosing unit and GRADIS dispersing unit, Sympatec GmbH, Clausthal-Zellerfeld, Germany). To avoid deviations caused by ungranulated primary particles, the size fraction from 250 to 500 μm was selected as representative granules. The sphericity of the granules was determined as a mean value (*n* = 3), with each sample containing at least 10^5^ particles. The sphericity is defined as the ratio of the perimeter of the area equivalent circle to the real perimeter.

Mercury Intrusion Porosimetry (PoreMaster®60GT, Quantachrome Instruments, Boynton Beach, FL, USA) was used to measure the pore size distribution and calculate the envelope volume *V*_*E*_ of the granule size fraction from 250 to 500 μm, which is the total volume occupied by the granules including the intragranular pores. It has to be considered, that for the calculation of the pore diameters or the pore size distribution, respectively, according to the Washburn equation, basic assumptions have to be made: The surface tension is constant at 0.485 N/m and the contact angle of the mercury during the measurement is 140°. Additionally, cylindrical pores are assumed, such that differently shaped pores cannot be recognised (Froboese et al., 2017).

Mercury intrusion porosimetry provides information about the entire sample porosity, i. e. inter- and intragranular porosity combined. However, for the calculation of the granules envelope volume, just the intragranular porosity is of importance. Thus, the inflection point of the cumulative intruded volume curve was defined as the threshold pore diameter, at which smaller pore diameter values were considered intragranular pores. Since only a defined size fraction of 250 to 500 μm of the granules was used for porosity measurements, the inflection point was distictive and therefore a reliable indicator for the shift from inter- to intragranular pores (León, 1998).

Further, helium gas pycnometry was applied for the determination of the true particle or granule density *ρ*_*true*_ (Ultrapyc 1200e, Quantachrome Instruments, Boynton Beach, FL, United States). By determining the granules envelope volume *V*_*E*_, the sample mass *m*_*S*_ and the true density *ρ*_*true*_, the porosity *ε* of the granules can be calculated (Webb, 2001):

(3) ε=1−ρEρtrue=1−mSVEρtrue

In order to visually assess the microstructure of the granules, scanning electron microscopy (SEM) images were taken (Helios G4 CX, Thermo Fisher Scientific, Hilsboro, OR, United States) after the samples were sputtered with platinum (LEICA EM ACE600, Leica Microsystems GmbH, Wetzlar, Germany).

#### 2.3.3 Residual moisture

The residual moisture of the wet granules as well as of the primary material was determined by loss on drying in the convective drying oven. Samples were dried at 80 °C for 24 h and wet and dry mass was compared.

Calculation of the theoretical residual moisture *RM*_*theo*_ was done by applying Eq. (2):

(4) $$RM_{theo}=\frac{RM_{MCC}+\left(\frac{L}{S}\right)\cdot \omega_{H_{2}O}}{1+\left(\frac{L}{S}\right)}$$

with $\omega_{H_{2}O}$ being the mass fraction of water in the binder liquid, *L/S* being the L/S-ratio and *RM*_*MCC*_ being the residual moisture of the primary material.

#### 2.3.4 Hausner ratio

A tapped density analyzer (Erich Tschacher Laboratoriumsbedarf, Bielefeld, Germany) was used to determine bulk density *ρ*_*bulk*_ and tapped density *ρ*_*tapped*_ of the raw material as well as the granulated powder. For each powder, three samples were measured and mean values (*n* = 3) were calculated. Hausner ratio *H* was applied to evaluate the flowability of the powders (Hausner, 1967):

(5) $$H=\frac{\rho_{tapped}}{\rho_{bulk}}$$

#### 2.3.5 Residence time distribution

Residence time distribution (RTD) of the granules was evaluated by conducting pulse response experiments. In these experiments, a tracer pulse is added to the system, approximating the shape of the Dirac delta function. 1 mL of an aqueous solution of brilliant blue FCF (20 mg/100 mL) was added directly into the binder solution stream just above the binder feed pipe into the granulator. Thus, disturbing effects on impulse shape of the tracer flow due to the flow regime in the tube conveying the binder solution were minimised. As mentioned in 2.2, a period of 1 min was given for the dry powder to form a ring layer in the granulator before starting the binder supply. Afterwards an additional 2 min were given for the granulation process to reach steady state. Thereafter, the tracer was injected (*t* = 0) and samples were collected every 5 s for up to 1 min, then every 10 s for another minute and then every 30 s for another 3 min. The obtained samples were dried as described in chapter 2.2. Every sample was dispersed in deionised water at a mass ratio of 1:10 and was stirred on a magnetic stirrer for 20 min to extract the brilliant blue from the granules. After 10 min of sedimentation, the supernatant was filtered through a 0.45 μm glass fibre filter to remove any excess solid particles in the liquid. Visible light absorption at 630 nm was measured by UV–vis-spectroscopy (Specord 210 PLUS, Analytik Jena GmbH + *Co*.KG, Jena, Germany).

In order to compare the RTD profiles of different granulation runs, the absorption profile *C(t)* was converted to the RTD function *E(t)* by normalising this profile by the area under the curve with *C(t)* being the absorption value for the respective sample taken at process time *t* (Engisch and Muzzio, 2016; Prziwara et al., 2021; Wen and Fan, 1975).

(6) $$E\left(t\right)=\frac{C\left(t\right)}{\int_{0}^{\infty }C\left(t\right)dt}$$

The mean residence time (MRT) *τ* is defined as the first moment of the RTD:

(7) $$\tau =\int_{0}^{\infty }t\cdot E\left(t\right)dt$$

with *t* being the mean of the time intervall the respective sample was collected.

Knowing the MRT and the solid mass feed rate *ṁ*_*S*_, the theoretical hold up mass *m*_*H*_ inside the granulator can be calculated:

(8) $$m_{H}=\tau \cdot {\dot{m}}_{S}$$

For better comparability of RTDs of the granulation runs and calculation of the axial dispersion, the dimensionless residence time *θ* is used:

(9) $$\theta =\frac{t}{\tau }$$

Thus the dimensionless RTD *E(θ)* is defined as:

(10) $$E\left(\theta \right)=\tau \cdot E\left(t\right)$$

The variance *σ*_*θ*_^*2*^ of the dimensionless RTDs *E(θ)* is calculated to determine the width of the distributions:

(11) $$\sigma_{\theta }^{2}=\int_{0}^{\infty }{\left(\theta -\bar{\theta }\right)}^{2}\cdot E\left(\theta \right)d\theta$$

Additionally, the axial dispersion model was applied, which characterises the convective and dispersive transport in continuous processes. Originating from the one dimensional Fokker-Planck-Equation, it was adapted for solids processing like blending or milling (Prziwara et al., 2021; Molerus, 1966; Sommer et al., 2013; Kwade, 1998; Sommer, 1977) and describes the change of the tracer concentration *c*_*T*_ over time:

(12) $$\frac{\partial c_{T}}{\partial \theta }=-\frac{\partial c_{T}}{\partial X}+\frac{1}{Pe}\frac{\partial^{2}c_{T}}{\partial X^{2}}\;$$

with *X* being the dimensionless axial location *X = x/L*. The Péclet number *Pe* describes the ratio of convective to dispersive transport and thus can be used to quantify axial dispersion in the process. Low Péclet numbers are indicating strong dispersive transport and thus back mixing, while for high Péclet numbers the process is approaching ideal plug flow behaviour with nearly no back mixing (Weinekötter and Reh, 1995). The Péclet number was obtained by applying the axial dispersion model on the dimensionless empirical RTD data. Since closed-closed boundary conditions are assumed for the ring layer granulation, meaning that dispersion of the tracer is occuring only within the boundaries of this process, there is no analytical solution for the partial differential eq. (11). Instead, a numerical approximation by using finite differences with Nelder-Mead optimisation was applied (Xu et al., 1991; Westerterp et al., 1984; Escotet-Espinoza et al., 2019).

#### 2.3.6 Binder distribution

Binder liquid distribution in the ring layer granulation process was assessed, similar to other publications, by staining the binder solution with a water-soluble dye. It is hypothesised, that the dye distribution is representative for the liquid distribution in the process and binder distribution in the dry granules, respectively (Meng, 2018; Smirani-Khayati et al., 2009). To assess the distribution of the binder in the powder after ring layer granulation, brilliant blue FCF was dissolved in the binder solution (13 mg/100 mL_Binder_). Granulation was conducted as described in chapter 2.2 with the coloured binder solution for chosen process parameters. Sieve analysis of the dry granules was carried out as described above while additionally using the mesh sizes 710 μm, 355 μm, 125 μm, 90 μm and 25 μm. Granules of the different sieve fractions were prepared for UV–vis-spectroscopy and absorption was measured as described above. Additionally, microscopic images of the granules were taken by reflected light microscopy (alpha300 R, WITec Wissenschaftliche Instrumente und Technologie GmbH, Ulm, Germany) for optical assessment of the binder distribution. Focus stacking was used at 20-fold magnification to create images of high resolution.

## 3 Results and discussion

### 3.1 Ring layer formation

In the ring layer granulator, the supplied particles are exposed to different kinds of forces, which are affecting the ring layer formation. On the one hand, gravitation is forcing the particles to move to the bottom. On the other hand, axial, radial and particularly tangential forces act on the powder or the individual particles, respectively, by the tools of the rotating shaft. Due to their movement on a circular path resulting from the tangential forces transferred by the tools of the shaft, the particles are exposed to high centrifugal forces and are accelerated towards the inner wall of the process chamber. In the case of the horizontally orientated ring layer granulator, these two forces mentioned above are partially working against each other. The ratio of centrifugal to gravitational force is described by the Froude number (Ramaker et al., 1998). Based on results for solids mixing processes, a threshold ratio of the Froude number has to be reached for a continuous ring layer to form at which centrifugal force superimposes weight force (Müller, 1981; Weinekötter and Gericke, 1995; Habermann, 2025).

When expressed in its quadratic form in eq. (12), the Froude number *Fr’* is dependent on the centrifugal acceleration *b*_*Z*_ and the gravitational acceleration *g*:

(13) $$Fr^{'}=\frac{b_{Z}}{g}=\frac{v_{t}^{2}}{r\cdot g}$$

with *v*_*t*_ being the tool tip speed and *r* being the radius of the shaft with tools.

In continuous horizontal blending processes, increasing Froude numbers lead to a certain degree of powder bed fluidisation, a more uniformly particle distribution and diminished effects of powder cohesion on the flow pattern (Sarkar and Wassgren, 2009; Sarkar and Wassgren, 2010). Generally, high Froude numbers indicate a force ratio such that the centrifugal force predominates the gravitational force. Consequently, the bulk powder forms a concentric, dynamic layer on the inner granulator chamber wall (Meng et al., 2016). However, it has to be taken into account, that the Froude number does not consider material properties. Thus, it is just an approximation to describe the real particle flow patterns. According to literature, the threshold Froude number at which ring layer formation occurs is varying between *Fr* > 7 and *Fr* > 11 (Müller, 1981; Weinekötter and Gericke, 1995; Habermann, 2025). For Froude numbers *Fr* > 1, at least at the outer tips of the tools centrifugal forces predominate gravitational force and the particles are thrown (Müller, 1981; Weinekötter and Gericke, 1995). Since the tool tip surface is significantly smaller than the surface of the inner granulator chamber wall, the collision frequency must be sufficiently high to form a stable ring layer. Based on the existing studies for ring layer granulation, a Froude number of *Fr* > 9 is assumed to be required for stable ring layer formation (Meng et al., 2016). Since all granulation runs were executed on the Granucon®1, there were no changes in geometrical dimensions. Subsequently, only the shaft rotational speed affects the ring layer formation. Thus, a minimal rotational speed *n*_*min*_ can be calculated below which insufficient ring layer formation, i. e. *Fr* < 9, is expected:

(14) $$n_{min}=\frac{9}{2\cdot \pi }\cdot \sqrt{\frac{g}{r}}$$

For the Froude number to exceed a value of 9, a minimal rotational speed of *n*_*min*_ = 1481 rpm, which translates to a tip speed of approximately 4.9 m/s, has to be reached. For the other tip speeds applied in this study, Froude numbers of 4.6, 13.7 and 18.2 were reached at 2.5 m/s, 7.5 m/s and 9.9 m/s, respectively.

### 3.2 Residence time distribution

Residence time is a crucial attribute of continuous processes since it determines the time, a material is exposed to the property changing impacts of the respective process. For a granulation process, a certain time is necessary for particle wetting, nucleation, growth and breakage of granules such that the specified granule properties are obtained (Iveson et al., 2001; Vonk et al., 1997).

RTDs of ring layer granulation runs with variation of tip speed, L/S-ratio or solid feed rate were conducted as described above. For each selected parameter, the other two parameters were held constant at the respective values of the reference run (4.9 m/s, 0.130 g_L_/g_S_, 2 kg/h). No runs were conducted at a tip speed of 2.5 m/s, because, as discussed later, no stable ring layer is formed at this speed, resulting in inacceptable granule size distributions.

**Fig. 2:**
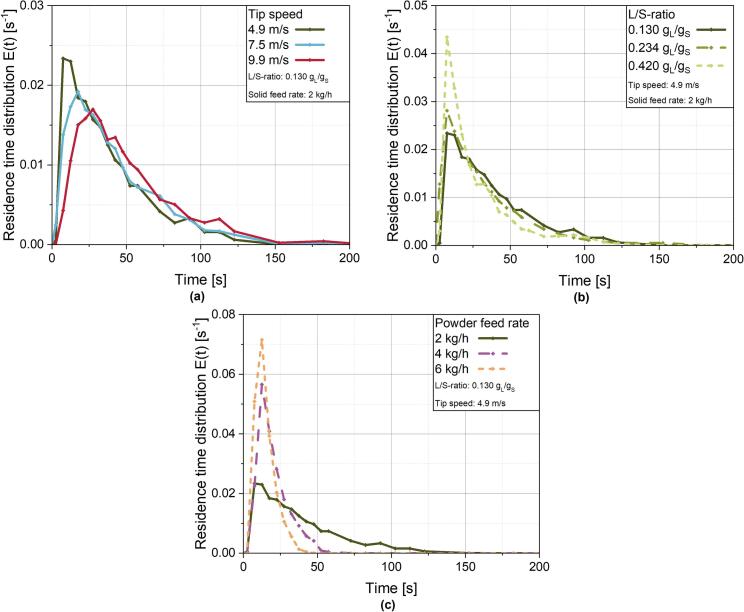
RTDs of the ring layer granulation at **(a)** different tip speeds, **(b)** different L/S-ratios and **(c)** different powder feed rates. Respective constant parameters for each group of runs are given in the legend.

For all evaluated RTDs displayed in Fig. 2 it can be seen, that the distributions are strongly asymmetrical. The highest values of the distributions are left of the mean value, which is in accordance with the observation, that most of the tracer is leaving the granulator a few seconds after addition. Additionally, there is a long tail of the RTDs.This is described by the variance *σ*^*2*^_*θ*_, which is a measure for the spread of the RTD (Rodrigues, 2021). Further, the skewness of the distributions was calculated and the values are given in the supplementary (Table S3).

In comparison to the variance of the RTD determined on a larger scale ring layer granulator of up to 0.44 (Meng et al., 2016), the variances measured in the present study are mostly higher with values up to 0.67. This suggests a more intense mixing in relation to the axial transport in the lab scale granulator. This is supported by the results for the Péclet number, which are relatively low and indicate a strong dispersive transport. Interestingly, with increasing shaft speed, the MRT is also increasing (Fig. 2a, Table. 2). This observation is contrary to some observations with similar devices, e. g. continuous blenders (Vanarase and Muzzio, 2011) or observations with other continuous granulation processes such as the twin screw granulation (Meng, 2018). In these processes, a decreasing MRT could be determined with increasing shaft speeds since a higher tool speed promotes axial powder transport. However, for continuous blending there is no ring layer formation and for twin screw granulation, granule transport is realised by screws fitted to the barrel dimensions and not by a rotating shaft with tools that provide a comparably large free volume towards the perimeter such that there is just a limited comparability of these processes with RLG. This leads to a decreased comparability of these processes with the ring layer process, in which centrifugal motion of the particles possesses a significantly larger impact on powder transport through the granulator. Thus, the increasing MRT with increasing shaft speed in the ring layer granulator is at least partly attributable to the ring layer itself. *Meng* et al. (Meng et al., 2016) could make similar observations for larger scale ring layer granulators. The increasing shaft speed leads to an increase in centrifugal forces and subsequently to a more pronounced tangential movement of the particles in the ring layer. A denser ring layer is formed, which presumably leads to a lower ring layer height and consequently to less particles forced directly by the tools in the axial direction. Consequently, axial movement to the granulator exit is reduced which results in prolonged MRTs. The higher value of the Péclet number underlines this assumption and indicates pronounced dispersion of the granules in the ring layer.

**Table 2:** Characteristic values of the RTDs under variation of tip speed, L/S-ratio and powder feed rate.

Parameter varied	Value	MRT *τ* [s]	*σ*^*2*^_*θ*_ [−]	Hold-Up [g_Gran_]	*Pe* [−]
Tip speed [m/s] *(L/S = 0.130 g*_*L*_*/g*_*S*_*and powder feed rate = 2 kg/h)*	4.9	37.68	0.54	20.93	1.07
	7.5	43.27	0.53	24.04	1.43
	9.9	52.51	0.50	29.17	2.32
L/S-ratio [g_L_/g_S_] *(tip speed = 4.9 m/s and powder feed rate = 2 kg/h)*	0.130	37.68	0.54	20.93	1.07
	0.234	29.70	0.57	16.50	0.82
	0.420	29.90	0.67	16.61	1.13
Powder feed rate [kg/h] *(L/S = 0.130 g*_*L*_*/g*_*S*_*and* *tip speed = 4.9 m/s)*	2	37.68	0.54	20.93	1.07
	4	20.14	0.26	22.37	5.99
	6	14.81	0.21	24.68	8.31

L/S: *Liquid-to-Solid ratio*, MRT *τ*: *Mean Residence Time*, *σ*^*2*^_*θ*_: *dimensionless variance of the distribution*, *Pe* = *Péclet number*

Against that, an increasing L/S-ratio from 0.130 to 0.234 g_L_/g_S_, which is realised by a higher binder supply rate, leads to a decrease of MRT (Fig. 2b, Table. 2*)*. Since higher L/S-ratios cause a pronounced granule growth, which will be discussed further in chapter 3.3.3, larger granules with improved flowability (Schlick-Hasper et al., 2022) are formed and transported through the granulator. Thus, on the one hand the axial movement through the granulator is enhanced and MRT of the granules is decreased. On the other hand, variance is increasing with increasing L/S-ratio, while for the Péclet number no clear trend is observable. Additionally, no further decrease of the MRT is detected between 0.234 and 0.420 g_L_/g_S_. This is an indicator for bypass of large granules through the granulator, as they have grown too large to be sufficiently captured between the tool tips and the chamber wall. Thus, larger granules are subjected to more intense axial transport, while smaller granules are captured in the ring layer.

The increase in powder supply rate also results in a decrease of MRT and additionally narrows the RTD significantly. The latter effect is especially detectable for the increase from 2 kg/h to 4 kg/h, which results in a reduction of the variance from 0.56 to 0.26. The increase in the powder feed rate initially leads to higher hold up masses in the granulator. However, hold up mass isn't increasing at the same rate as the powder feed, which ultimately leads to a faster axial displacement of the powder in the ring layer. Thus, the mean residence time is reduced and its distribution is narrowed. Consequently, the calculated Péclet number is strongly increasing, emphasising the pronounced axial displacement with less back mixing. In studies about continuous powder blending, comparable results were obtained (Vanarase and Muzzio, 2011; Dubey et al., 2012; Lücke and Merz, 1977).

### 3.3 Effect of process parameters on the granule properties

#### 3.3.1 Influence of mechanical stress on dry powder

**Fig. 3:**
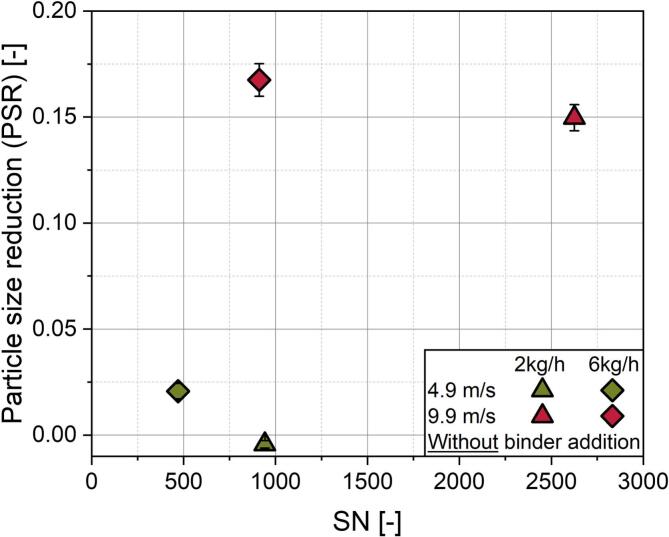
Influence of mechanical stress on dry MCC in the ring layer granulator. The effect is quantified by the particle size reduction and stress number is approximated with MRT at the respective parameter settings and shaft speed.

Granulation processes consist of particle agglomeration, but also agglomerate breakage due to mechanical stress. Both events are occurring simultaneously throughout the process and shape the final size distribution of the granules (Iveson and Litster, 1998; Ramaker et al., 1998; Tardos et al., 1997). To assess the mechanical stress acting on the powder particles in the ring layer granulator, the process was conducted in the absence of any binder liquid to avoid any cohesive forces due to liquid bridging and by that also agglomeration. Different parameter settings of the tip speed and solid feed rate were investigated, of which both are affecting the hold up mass in the granulator. The particle size reduction (PSR, change of the mean particle size in relation to the original mean particle size, Fig. 3) can be seen as a surrogate for the input of mechanical stress. With higher speed of the shaft it can be assumed, that more stress is exerted on the particles, which leads to a more pronounced PSR. In order to theoretically quantify the number of stress events (SN) which act on the ring layer, it is approximated by multiplying the MRT *τ* with the shaft revolution frequency *n*:

(15) SN=τ·n

Only for the particles stressed at 2 kg/h and 4.9 m/s, no reduction of the mean particle size could be observed, which is presumably due to insufficient stress intensity at such low shaft speeds in combination with the sparsely filled ring layer. Interestingly at solid feed rates of 6 kg/h, although resulting in lower number of stress events than at 2 kg/h due to the significantly decreased MRTs, the PSR is still higher at both shaft speed settings. This can be attributed to the larger hold up mass in the granulator chamber at higher solid feed rates. An enhanced fill level presumeably leads to a higher integrated centrifugal load and thus to a densification of the ring layer due to the higher rotating mass. Consequently, more particles are subjected to potentially size reducing events with every pin passing in combination with higher centrifugal load. Additionally, stress can be transmitted more effectively, because the formation probability of force chains across particles being compressed between the shaft tooling and the process chamber wall is increasing with increasing fill level and ring layer densification, ultimately leading to an enhanced PSR.

#### 3.3.2 Binder distribution in ring layer granulation

**Fig. 4:**
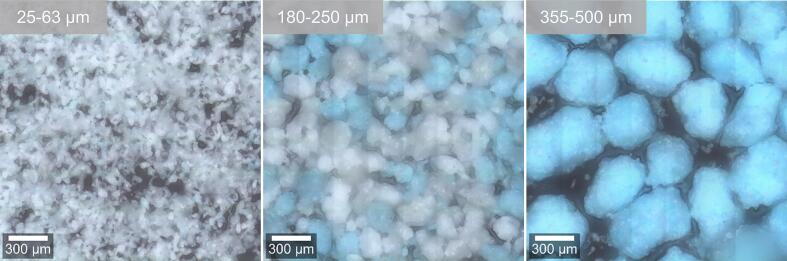
Reflected light microscopic images of granules to evaluate the distribution of coloured binder in ring layer granulation. Numbers the top left corners show the sieve fractions. Granules were produced at a tip speed of 4.9 m/s and an L/S-ratio of 0.234 g_L_/g_S_.

The distribution of the binder solution is crucial for homogeneous granule growth and has been investigated in several studies for batch high shear granulation (Smirani-Khayati et al., 2009; Vandevivere et al., 2020; El Hagrasy et al., 2013). In Fig. 4, the binder distribution for the ring layer granulation considered in the present work can be evaluated directly by the particle colouring. Herein it is assumed, that particles showing colouration were sufficiently wetted by the stained binder solution and thus were able to form granules.

Different intensities of colouration can be observed for different particle size fractions. For the smallest particle sizes (25–63 μm), for which no agglomeration occurred with reasonable assumption, interestingly a slight degree of colouring can be seen. This can either be attributed to small particles, to which part of the dye was transferred to by contact with larger granules without integration into the granule, or to attrition of larger granules such that dyed fragments appear at low particle sizes. Similar observations were made for batch high shear granulation, for which granule breakage was concluded as the main cause of colouration of particles with sizes in the range of the primary particles (Westerterp et al., 1984).

For the size fraction of 180–250 μm it can clearly be seen, that the binder distribution is inhomogenous. Coloured granules as well as uncoloured primary particles are existent in this size fraction. This suggests that a part of the primary particles leave the granulator without being wetted and granulated. For the size fraction larger than 355 μm, only coloured granules are present, because most primary powder particles are smaller than that with an $x_{90,3}$ value measured by laser diffraction (LD) of $x_{90,3,\mathrm{LD}}$ = 173 μm.

**Fig. 5:**
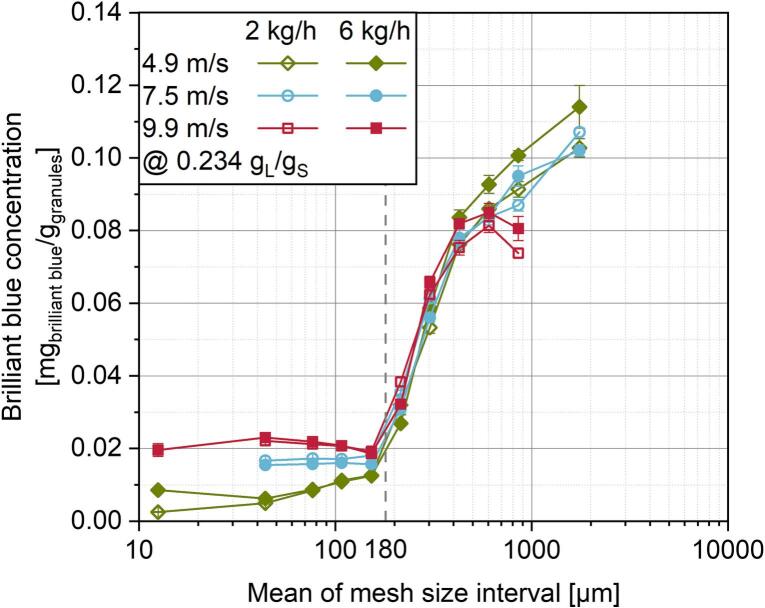
Binder content in the granules determined by the surrogate of the concentration of brilliant blue as a function of granule size fractions at a constant L/S ratio.

In Fig. 5, the binder distribution in different granule size fractions is described quantitatively as a function of shaft speed and solid feed rate. Analogous to the paragraph above, it is assumed, that the share of brilliant blue is directly proportional to the degree of wetting of the powder in the granulator and thus can be linked to the binder proportion in the respective granules. Additionally, a threshold particle size was defined at 180 μm, above which the powder was considered granulated. This will be discussed in further detail in chapter 3.3.3. The share of brilliant blue and thus the binder proportion in the granules is generally low for sieve fractions below the threshold value of 180 μm. As already seen in Fig. 4, a certain amount of brilliant blue can be detected for the smallest particle sizes. Here, the assumption of attrition and granule breakage is supported by the different shares of the dye at varying shaft speeds and thus at varying input of mechanical stress as already described in chapter 3.3.1. With increasing shaft speed, the share of brilliant blue is increasing in the size range below 180 μm due to higher mechanical stress. Granule breakage is more pronounced, which leads to higher shares of coloured fragments in the small size fractions. At further rising particle sizes up to the threshold of 180 μm, different behaviour can be observed for the different shaft speeds. For low speeds at 4.9 m/s, the share of brilliant blue is rising with increasing granule size fraction, for 7.5 m/s there is no significant change and for 9.9 m/s the share is even slightly declining again. These observations may also be attributable to the varying input of mechanical stress: At higher speeds and thus higher stress intensities, granules break into smaller fragments than at lowest speed, which results in the detection of more dye in smaller size fractions. For the lower speeds, however, additional to the overall lower share of granule fragments, the fragments resulting from breakage are generally coarser. This leads to the increasing share of dye with increasing granule size fractions in the range between 50 μm and 180 μm.

At particle sizes above the threshold of 180 μm, the binder proportion is strongly increasing with increasing granule sizes. This is expectable to a certain degree, because based on the $x_{90,3,\mathrm{LD}}$ of the primary powder, the share of uncoloured und thus ungranulated primary particles in those size fractions is strongly decreasing. Apart from that, even at coarser size fractions above 355 μm, in which only granules are present, an increasing binder content with increasing granule size can be observed (Fig. 5). This is because higher degrees of local particle wetting are necessary for larger granules to form. Thus, the concentration of binder in those granules is generally higher. Interestingly, for granules larger than 400 μm, the binder share is lower for higher shaft speeds.

This can probably be traced back to the influence of intensified breakage events at higher shaft speeds. Granule growth by addition of dry primary particles happens as long as sufficiently wetted granule surface is available. As a consequence of granule breakage, wetted fracture surface is exposed, to which subsequently uncoloured fine particles can adhere. In return, specific binder content is decreasing. Additionally, longer residence times enhance this effect. Thus, the binder is overall distributed to more particles and the binder share for single granules is reduced. Further, a significant difference in the share of brilliant blue between the varying solid feed rates is prominent for granule fractions of larger sizes. As residence time is considerably shorter for higher solid feed rates, there is less time for the binder liquid to be distributed homogeneously in the ring layer and for coarser granules to break. Thus, the binder content is increased for higher solid feed rates at otherwise equal L/S-ratios and shaft speeds.

The overall binder distribution as a function of the granule size shown in Fig. 5 is very similar to the binder distribution in conventional batch high shear granulation processes from other studies and indicates an inhomogeneous distribution (Smirani-Khayati et al., 2009; Reynolds et al., 2004; Ax et al., 2008; Knight et al., 1998; Scott et al., 2000). When comparing the results in the present work with results from literature, the assumption arises, that the binder distribution shown here corresponds with that of batch high shear granulation directly after liquid addition or very short processing times, respectively. While for longer wet massing times in batch processes, there is a more or less pronounced homogenisation of the binder distribution (Smirani-Khayati et al., 2009; Reynolds et al., 2004; Knight et al., 1998), this effect cannot be observed here for continuous RLG. This is presumably caused by the short residence times of the ring layer granulation runs. Although significantly higher shaft speeds are applied in RLG than impeller speeds in batch processes, which potentially lead to a more uniform liquid distribution (Smirani-Khayati et al., 2009; Litster et al., 2002), there is not enough time under mechanical stress to sufficiently homogenise the binder liquid distribution. Contrary to the present results, however, *Meng* et al. (Meng et al., 2016) obtained a homogeneous liquid distribution over all granule size fractions in the larger scale ring layer granulator similar to that of batch high shear processes after longer wet massing times, although the residence time is even lower than in the present work. This indicates quite significant differences in the granulation process between the new lab scale and the larger scale ring layer granulator. This may be attributed to the different granulator diameters. For the larger ring layer granulator, due to the nearly doubled diameter, shaft circumferential velocities are significantly higher at the same rotational speeds, such that the powder is transported much faster through the wetting zone, but is also exerted to higher impact and compressive stresses. Consequently, binder liquid drops are exposed to a larger volume of powder and can be distributed more homogeneously (Litster et al., 2001). As for the lab scale granulator, the maximum adjustable speed is already reached at 9.9 m/s in the present study, such high speeds could not be reproduced. Accordingly, further investigations regarding the effect of the granulator geometry, scale and of different formulations should be conducted to deepend systematic knowledge.

#### 3.3.3 Influence of process parameters on final granule size

**Fig. 6:**
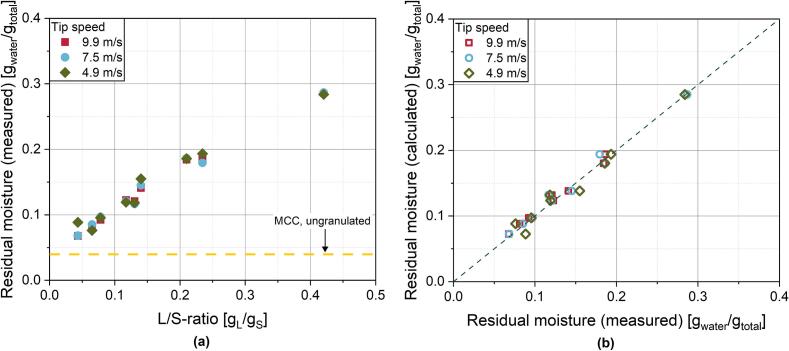
**(a)** Residual moisture content of the RLG granules as a function of L/S-ratio at varying shaft speeds and **(b)** parity plot of the measured and the calculated theoretical residual moisture of RLG granules.

**Fig. 7:**
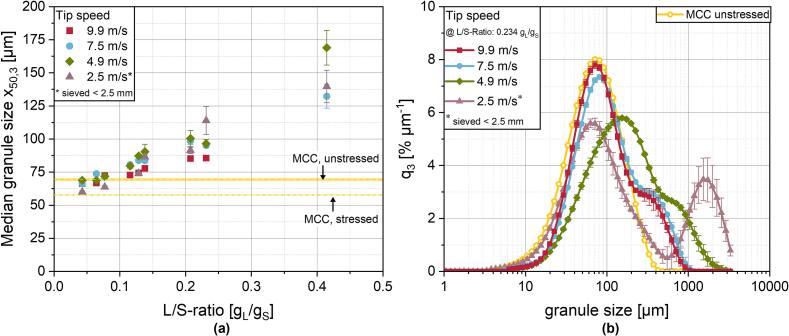
***(a)** Median granule sizes determined by laser diffraction. The yellow lines indicate the median particle size of MCC (unstressed and stressed [9.9 m/s, 6 kg/h] as described in chapter 3.3.1). **(b)** Exemplary granule size distributions for different shaft speeds at a constant L/S-ratio of 0.234 g*_*L*_*/g*_*S*_*. To support this explanation, the median particle size of the stressed MCC is given in*Fig. 7a, *which is smaller than those of the granules at low L/S-ratios.*

The L/S-ratio and thus the degree of moisture is usually the most important parameter in wet granulation regarding granule growth rate. As more water is supplied to the granulator, residual moisture of the granulated powder is rising with increasing L/S-ratio (Fig. 6a). Due to the higher moisture content and consequently improved availability of liquid on the particle surface, the formation of liquid bridges between the powder particles and thus the agglomeration tendency is promoted. Additionally, the higher share of binder introduced to the granules prevents deagglomeration to a certain degree (Iveson et al., 2001; Iveson and Litster, 1998). The resulting effect on the median granule size is displayed in Fig. 7. It is also evident that the residual moisture is unaffected by the shaft speed of the granulator (Fig. 6a). The parity plot (Fig. 6b) further shows good correlation of the measured residual moisture after granulation with the theoretical moisture calculated from the L/S-ratio, thus allowing to employ the L/S-ratio as a precise indicator for the final granule moisture.

It has to be mentioned, that at a tip speed of 9.9 m/s and an L/S-ratio of 0.420 g_L_/g_S_, the granulation couldn't be conducted as the granulator was clogged by local overwetting of the powder at the liquid feed location. This effect can presumably be attributed to the densification of the ring layer at high shaft speeds because of the enhanced centrifugal forces. Due to the lower porosity of the ring layer and the agglomerates in it (shown in chapter 3.3.4), complete liquid saturation of the powder bed is achieved already at lower L/S-ratios. Consequently, as binder liquid cannot further penetrate the densified ring layer, drop coalescence and subsequently oversaturation occurs at high L/S-ratios. This leads to the formation of an exponentially growing lump around the liquid feed location and clogging of the granulator, because the added liquid cannot be removed and powder cannot be transported further (Iveson and Litster, 1998; Knight et al., 1998; Vromans et al., 1999). For the lowest L/S-ratios, the median granule size falls below the median particle size of the starting material. Due to the insufficient wetting of the powder, the agglomeration tendency is lower than the impact of the mechanical stress exerted on the particles. By considering the conditions of the granulation process, namely the mechanical stress from granulator tools rotation and granule pore saturation with binder liquid, assumptions can be made about the granule growth behaviour based on the growth regime map for wet granulation proposed by Iveson and Litster (Iveson and Litster, 1998). Mechanical stress is generally quite high for ring layer granulation due to the high rotational speeds of the shaft. In combination with low L/S-ratios or low pore saturation, respectively, the granulation behaviour is located in the dry, free flowing powder regime. Consequently, no granule nucleation or growth is occurring (Iveson and Litster, 1998; Meng et al., 2016). Regarding the influence of the shaft speed in a range from 4.9 m/s to 9.9 m/s, it can be seen that at lower speed, generally larger median granule sizes were obtained. For the present work, this can be explained by the tendency for the granules to break, which is reduced by the decreased input of mechanical stress as a result of the slower shaft speed and shorter MRT (Ramaker et al., 1998). In comparison, the increase in median granule size with decreasing shaft speed is contrary to the results by *Meng* et al. (Meng et al., 2016) and *Järvinen* et al. (Järvinen et al., 2015), who observed larger median granule sizes with increasing shaft speeds in a larger scale ring layer granulator. A possible explanation can again be found in differing process conditions to the present work. *Meng* et al. applied L/S-ratios in similar orders of magnitude as in the present work, but reached significantly higher circumferential speeds of the shaft. The higher speeds lead to pronounced granule consolidation, which in return increases the maximum pore saturation with liquid and the granule strength. This subsequently leads to a rise of the growth rate (Iveson and Litster, 1998; Saleh et al., 2005), which predominates the breakage from the impact of mechanical stress. This effect is then intensified by further increasing shaft speeds (Meng et al., 2016). Although *Järvinen* et al. applied tip speeds in a similar range to the present work, the L/S-ratio was significantly higher with values of 0.66 to 0.79 (Järvinen et al., 2015). Again, the higher pore saturation, here due to an overall larger amount of available liquid, seems to predominate the granule breakage and an increase in shaft speed leads to an increase in median particle size.

The trend of increasing median granule sizes with decreasing shaft speeds is only observable for tip speeds down to 4.9 m/s. At 2.5 m/s, median granule sizes are located between the values for higher shaft speeds (Fig. 7a) which is presumably the result of insufficient ring layer formation as the shaft speed is not high enough to form a stable ring layer (see chapter 3.1). A more profound consequence of the lack of ring layer formation can be observed for the corresponding GSD (Fig. 7b). There, a distinct bimodal distribution with a high fraction of fine powder and a high fraction of overly agglomerated powder develops. This is the result of a lack of binder distribution, which was already discussed for higher shaft speeds in the previous chapter and is even more pronounced here. As the binder liquid drops initially form large lumps upon penetration into the powder bed, a bimodal particle distribution arises. This was found to be inevitable for high shear granulation processes, especially for liquid supply by pour on, as an instantaneous, homogeneous distribution of the binder liquid is physically impossible (Knight et al., 1998). As a result of the insufficient mechanical mixing at such low shaft speeds and the absence of the ring layer, no breakage of these large lumps occurs. Regarding the overall low residence times in the ring layer granulator, the observed effects at 2.5 m/s are comparable to results of batch high shear granulation at low impeller speeds and short process times. For these process conditions, a pronounced bimodal nature of the distributions were identified, which could be attributed to insufficient binder distribution due to low mechanical dispersion and short process times (Scott et al., 2000; Wildeboer et al., 2005; Knight et al., 2000).

**Fig. 8:**
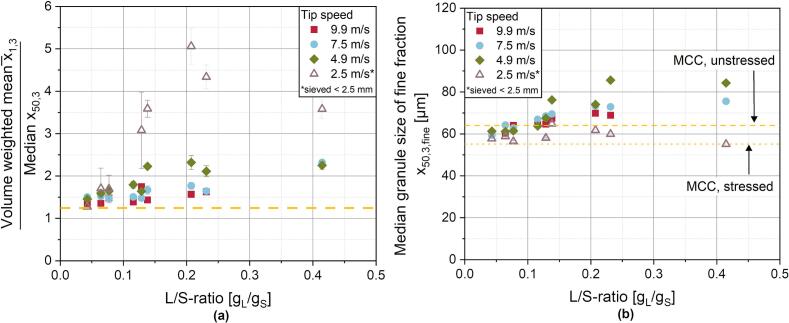
***(a)** Ratio of the volume weighted mean*${\bar{x}}_{1,3}$*to the median granule size*$x_{50,3}$*for each granulation run as a descriptive factor for the asymmetrical nature of the GSD. **(b)** Median granule size*$x_{50,3,\mathrm{fine}}$*of the fine fraction (< 180* μ*m) of the GSD as well as the unstressed and stressed primary powder. The corresponding values of the starting material are shown as yellow lines.*

Since the bimodal nature of the distributions cannot be described by the median granule size alone, an approach was chosen to describe the asymmetry of the distributions as a surrogate valueby relating the volume weighted mean granule size ${\bar{x}}_{1,3,\mathrm{LD}}$ to the median granule size $x_{50,3}$ of the distributions (Fig. 8a). It is assumend, that the closer the two values and thus, the quotient to 1, are, the less pronounced is the asymmetry of the distribution. Since larger granules lead to a more pronounced shift of the volume weighted mean than of the median value, stronger differences between the modal values lead to higher ratios of this quotient. As a result, this quotient incorporates a weighting of larger granules into the asymmetry and can be used as an initial indicator for the bimodal nature of the size distribution in this specific case. However, it has to be noted, that this approach for now is limited to the present results, in which a poor binder liquid distribution with primary material remaining in the product is present and the behaviour of the resulting size distributions is correctly displayed. This mean-to-median ratio has some limitations, when the distribution becomes symmetrical. This case is very improbable for the RLG process, but still it is recommended to use this value in combination with the resulting size distribution. For the lowest tip speed of 2.5 m/s, the highest ratios are obtained especially for increasing L/S-ratios as a result of poor binder distribution. However, the decrease at 0.23 and 0.42 g_L_/g_S_ is attributable to the sieving before the measurements, removing large lumps and a further increase of the ratio would be expectable. At tip speeds above 4.9 m/s, for which stable ring layer formation is assumed, the mean-to-median ratio is lower.

Still, a slight trend is observable with higher size ratios at lower tip speeds. At these tip speeds above 4.9 m/s, granule growth is mostly indicated by a shoulder in the particle size distributions, which is also reflected in a slight increase of the size ratio in Fig. 8a.

For the fine fraction (< 180 μm), the GSDs at 2.5, 7.5 and 9.9 m/s are otherwise similar to the primary particle size distribution. This is displayed by the median values of the granules fine fraction in Fig. 8b. Analogous to Fig. 7a, the fine fraction median values of the unstressed and stressed primary material is given, as the comminution effect described in chapter 3.3.1 is also observable. Interestingly, for the powder processed at 2.5 m/s even at high L/S-ratios, the effect is more pronounced than for higher tip speeds, which presumably is attributable to two effects originating from the insufficient ring layer formation: On the one hand, due to the limited distribution of the powder in the granulator and thus a concentration of the powder at the bottom of the granulator, the formation of force chains is pronounced when the tools run through the powder and the degree of comminution is higher. On the other hand, binder distribution is strongly inhomogeneuous and large lumps of wetted powder are not broken down. As a result, less granule nuclei and small fragments are created, which would result in higher median values in the fine fraction.

Overall, the median values of the fine fractions are just slightly increasing above the primary median particle size. This is again an indicator for the poor binder distribution and the presence of a large amount of primary particles after the granulation runs. This is also reflected in the Hausner ratios calculated for the resulting granules. As expected, Hausner ratio is decreasing with increasing L/S-ratio due to higher mass fractions of larger granules, indicating an improved flowability. However, as a result of the broadened size distributions, for lower L/S-ratios the Hausner ratio is higher than for the primary powder. Further details can be found in the supplementary (Fig. S4).

In addition to the particle size measurement by laser diffraction, sieve analysis of the granulated powders was performed. This allows for quantitative statements about the actual sufficiently granulated mass fraction of the powders. Analogous to previous sections, a threshold of 180 μm was defined, which corresponds to the $x_{90,3,\mathrm{LD}}$ value of the primary MCC. All particles above this size were considered as granulated.

**Fig. 9:**
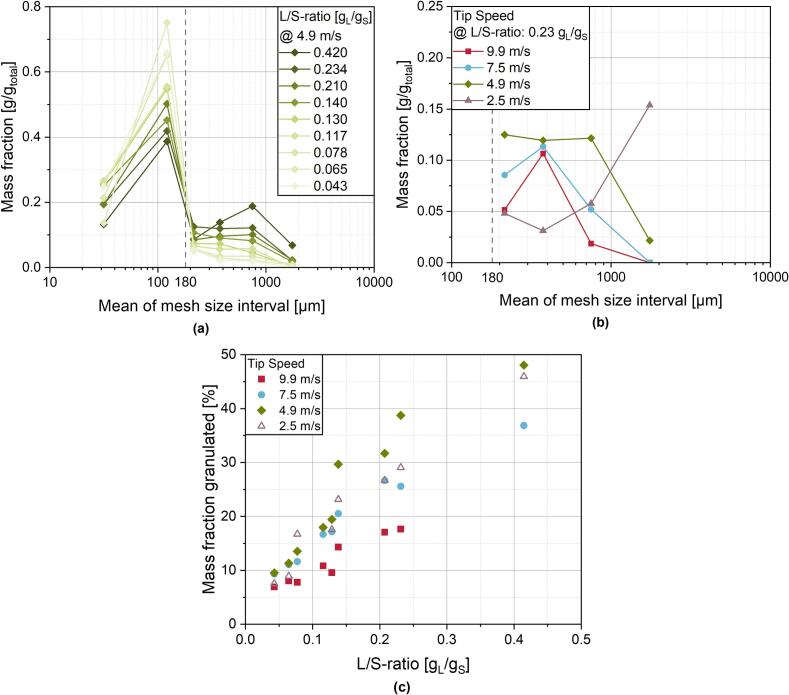
**(a)** Granule mass fractions obtained by sieve analysis for varying L/S-ratios at a constant shaft speed of 4.9 m/s. Above the threshold of 180 μm, successful granulation is assumed. **(b)** Effect of shaft speed on the granule mass fractions (above 180 μm) at a constant L/S-ratio of 0.234 g_L_/g_S_. **(c)** Mass fraction of granules (above 180 μm) as a function of the L/S-ratio with different shaft speeds.

As can be seen from the mass distribution of the sieve fractions at the exemplary tip speed of 4.9 m/s (Fig. 9a), with increasing L/S-ratios there is a steady increase in the mass of the sieve fractions above 180 μm while continuously reducing the mass fractions of fine powder below 180 μm. This is again indicating a pronounced agglomeration tendency at higher L/S-ratios up to an overall granulated proportion of 50 % at highest L/S-ratio employed (Fig. 9c). Moreover, at increasing tip speeds (Fig. 9b) a shift of the mass fractions to narrower distributions is visible. While for 4.9 m/s, coarse granules over 1000 μm were obtained, the fraction of coarse granules is decreasing with increasing shaft speeds. Because of the higher input of mechanical stress at higher shaft speeds, a more homogeneous distribution of the binder liquid is possible and thus a more homogeneous growth of the granules. Additionally, coarser granules broke down again in a larger extent narrowing the distribution.

The more pronounced breakage also results in smaller mass fractions of granulated material as the granules broke down to fragments below 180 μm (Fig. 9b, c).As already discussed above, at 2.5 m/s there is no complete ring layer formation in the granulator. At higher shaft speeds, at which a stable ring layer is formed, the proportion of large granules over 1000 μm is relatively low or even non-existent. On the contrary, there is a strong increase of granules exceeding 1000 μm at 2.5 m/s while the proportion of moderately sized granules is comparably low (Fig. 9b*)*. Actually, large clumps exceeding 1 cm in diameter occurred, which are not explicitly visible here, because the largest sieve mesh size was 1000 μm. Consequently, the overall mass fraction of granulated powder is raised by only a few large clumps with high masses, such that the granulation success at 2.5 m/s is shown in Fig. 9c with open symbols due to poor comparability with the other shaft speeds.

For the mass distributions in Fig. 9a as well as the granulation success, depicted as the granulated mass fraction in Fig. 9c, the large fraction of ungranulated fines is noticeable. Even for high L/S-ratios up to 0.41 g_L_/g_S_ only somewhat less than 50 % of the powder mass is granulated. As explained above, this is mostly caused by suboptimal binder liquid distribution even if the ring layer formation is successful. Additionally, it can be assumed from Fig. 3 and Fig. 4 that granule breakage and attrition also contribute to a reduced granulated mass fraction to a certain degree. In studies regarding a larger scale ring layer granulator, such high fractions of fines were not obtained at similar L/S-ratios (Järvinen et al., 2015; Meng et al., 2016). However, in the study from *Meng* et al. (Meng et al., 2016), next to the already discussed difference in tip speeds, the binder was dry mixed with the powder formulation before and distilled water was used as the granulation liquid. This may be beneficial for the granulation in the ring layer, as the binder is already homogeneously distributed in the powder and water with lower viscosity than an aqueous solution of the binder has superior spreadability and leads to an enhanced liquid distribution (Hapgood et al., 2003).

Additionally, lactose was used in the formulation, which possesses a different wetting behaviour than MCC. For MCC, more water is necessary to form surface water on the particles and thus liquid bridges between particles than for lactose, which needs to be available for successful granulation. Shortly after adding the binder solution, the water is absorbed by the MCC particles in immediate vicinity, such that there isn't enough surface water available for the remaining particles throughout the further granulation process to sufficiently agglomerate (Iveson and Litster, 1998; Chitu et al., 2011a; Chitu et al., 2011b; Kleinebudde, 1997). On the one hand, adding a higher amount of binder liquid can potentially decrease this effect. On the other hand, due to clogging of the granulator already observed in the present work at high L/S-ratios, a more intense initial dispersion of the liquid is necessary for the ring layer granulator. From an industrial and sustainability perspective, adding more water is also accompanied by longer drying times with higher energy comsumption.

#### 3.3.4 Influence of process parameters on the morphology of ring layer granules

**Fig. 10:**
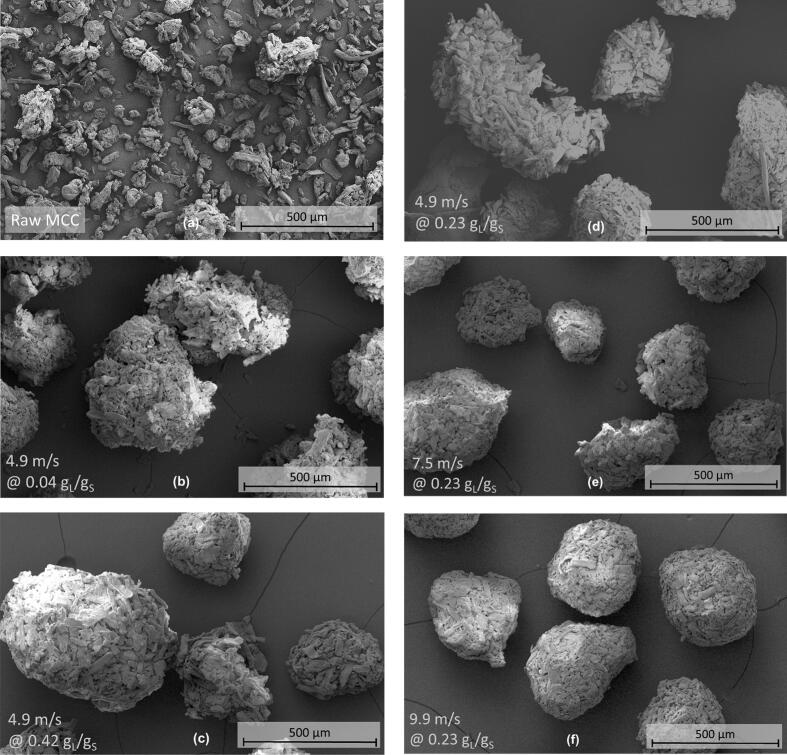
SEM images of raw MCC and ring layer granules for different RLG parameter settings. **(a)** MCC primary material, **(b,d,c)** increasing L/S-ratios at a constant tip speed of 4.9 m/s and **(d)-(f)** Increasing tip speed at a constant L/S-ratio of 0.234 g_L_/g_S_. For the images of granules, sieve fractions from 250 μm to 500 μm are used.

The morphology of granules is of great importance, as it influences the powder flowability and tabletability, among other things (Fu et al., 2012; Hancock, 2019; Johansson and Alderborn, 2001). In Fig. 10*,* the effect of various process parameters on the morphology can be observed on the basis of individual granules. With rising shaft speeds at a constant L/S-ratio (Fig. 10d-f), it is apparent that granules become more spherical and dense. This observation can also be quantified by the shape factor of sphericity (Table. 3).

**Table 3:** Sphericity and porosity of ring layer granules (sieve fraction of 250–500 μm) for varying granulation parameter settings to quantitatively describe the morphology of the granules.

Tip speed [m/s]	L/S-ratio [g_L_/g_S_]	Sphericity [−]	Porosity *ε* [−]
4.9	0.234	0.813 ± 0.003	0.535
7.5	0.234	0.845 ± 0.001	0.499
9.9	0.234	0.851 ± 0.001	0.471
4.9	0.043	0.806 ± 0.002	0.535
4.9	0.234	0.813 ± 0.003	0.535
4.9	0.420	0.821 ± 0.002	0.489

For sphericity values approaching the value 1, granule shape approaches the shape of an ideal sphere. Combining the results of the sphericity measurement and the SEM images, granules from ring layer granulation are in general quite spherical and compact with granule sphericity values above 0.8. The stress exerted on the granules by the high shear rates in the ring layer process lead to pronounced particle rounding and densification. Especially for high tip speeds, i. e. higher shear rates and mechanical stress, plastic granule deformation can be assumed from SEM images whereas the primary particles are forced into intense contact. For 9.9 m/s in Fig. 10f, the granule surface is nearly sealed by the primary particles, while at 4.9 m/s in Fig. 10d granule surface is more porous and single primary particles, with similar morphology to the raw material in Fig. 10a sticking out, are roughening the surface. This leads to a positive correlation of granule sphericity with the selected shaft speed (Table. 3).

**Fig. 11:**
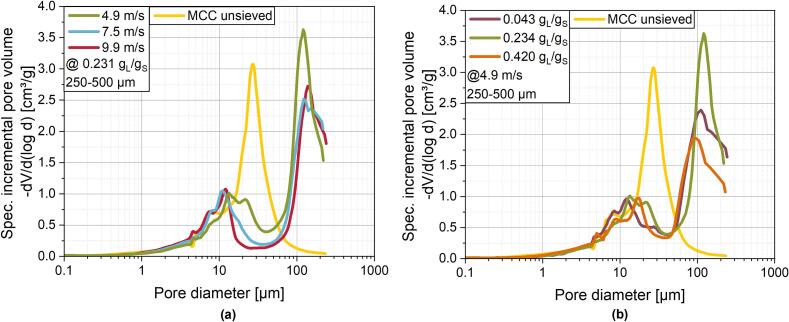
Pore size distributions for ring layer granules and ungranulated MCC determined by mercury porosimetry for size fractions of 250 μm to 500 μm. **(a)** Granules made at varying shaft speeds and a constant L/S-ratio of 0.234 g_L_/g_S_ and **(b)** at varying L/S-ratios at a constant shaft speed of 4.9 m/s.

For high L/S-ratios, the sphericity is also higher than for low L/S-ratios. An increase in water content and thus binder content in the granules is promoting granule deformability without particle breakage as it decreases the interparticle friction (Iveson et al., 1996). Additionally, improved cohesion can be assumed due to higher binder contents on the granule surface, which leads to decreased protrusion of primary particles. Similar results have been observed in batch high shear processes with increasing impeller tip speed (Ohno et al., 2007) as well as in larger scale continuous ring layer granulation with increasing shaft speed and L/S-ratio (Järvinen et al., 2015; Meng et al., 2016). A closer look on the microstructure of the granules is given by the overall intra-granular porosity (Table. 3) and pore size distributions (Fig. 11).

Regarding the pore size distributions, it has to be taken into account, that for the granules the sieve fraction of 250–500 μm was investigated to give a clear distinction between inter- and intra-granular pores while for the primary particles the unsieved MCC was used to consider all primary particles of which the granules are made of. The values for intra-granular porosity in Table. 3 are determined based on the envelope density of the granules calculated from mercury intrusion experiments, thus inter-granular porosity is neglected. Intra-granular porosity is in the range of 0.471 to 0.535, which is in accordance with results from *Meng* et al., who obtained intra-granular porosities in the range of about 0.27 to 0.63 in their ring layer process (Meng et al., 2016). However, significantly lower porosities with values down to 0.12 could be created in high shear granulation processes for MCC granules (Benali et al., 2009), presumably due to longer wet massing times in the batch process compared with continuous RLG, which leads to increased granule consolidation. With increasing tip speeds at constant L/S-ratio, the overall intra-granular porosity is decreasing from 0.535 to 0.471 (Table. 3) due to densification as a result of higher shear and compression stresses as described above. Additionally, there is a shift observable in the pore size distribution in the range of about 20–30 μm. There, the occurrence of larger pores is diminishing with increasing shaft speed. As a result of the more intense mechanical stress at high shaft speeds, granules densify by means of closing larger pores due to particle rearrangement within the granule and plastic particle deformation. For pore sizes below 10 μm, no such clear trend can be determined. However, granule pore size distribution in this range is similar to the primary particle pore size distribution. Thus, it can be assumed, that the mechanical stress is not sufficient to significantly alter the pores between fine primary particles. Increasing L/S-ratios also lead tendency-wise to a decrease in intra-granular porosity from 0.535 to 0.489. This can again be attributed to the improved deformability of the granules with higher liquid content (Knight et al., 1998; Bouwman et al., 2005), thus closing pores even at low shaft speeds. Interestingly, porosity values of the granules made with an L/S-ratio of 0.043 g_L_/g_S_ and with an L/S-ratio of 0.234 g_L_/g_S_ remain the same while clear differences can be seen in the pore size distribution. Contrary to the expectations of lowering the pore diameters, for the L/S-ratios of 0.420 and 0.234 g_L_/g_S_ there is a more frequent occurrence of larger pores than for 0.043 g_L_/g_S_. This may be explained by the low degree of granulation for the latter set of parameters as already shown in the previous chapter (Fig. 9c). As a result, a larger fraction of ungranulated primary particles of this size than granules are present in the sieve fraction. Porosity measurements showed, that pore diameters of the primary particles are smaller than for the granules, even if the same sieve fraction is analysed. This leads to an overall less frequent presence of larger pores for very low L/S-ratios. However, it has to be considered, that for the determination of the pore size distribution by mercury intrusion porosimetry, certain assumptions need to be made, which do not necessarily reflect the real conditions like the occurrence of differently shaped pores, e. g. bottleneck pores. To prove the above mentioned presumptions, further investigations regarding the formation of the pore size distributions and pore shapes in RLG processes should be conducted.

## 4 Conclusion

The influence of varying process parameters on the resulting granule properties in a continuous wet granulation process using a ring layer granulator at novel lab scale was evaluated. This is the first time results of the ring layer granulation in such a small scale are reported. In comparison with larger scale RLG, similar trends in the results of the wet granulation were observed regarding residence time behaviour as well as granule shape and porosity. In addition, the L/S-ratio was observed to be the most influential parameter regarding the final granule size and with increasing levels could produce larger granules. However, poor binder distribution of the actual configuration led to smaller mean granule sizes, more bimodal granules size distributions and an overall lower mass fraction of granules compared to the larger scale process as reported in literature.

Main reason beside the poor binder distribution was the use of only microcrystalline cellulose as an insoluble primary material. On the one hand, it allowed the application of a wide range of parameter settings due to its robust wet granulation behaviour. On the other hand, it led to the mentioned challenges as a result of its water absorption properties in combination with the binder supply in dripping mode and the short residence times. Consequently, MCC as a single component of the primary powder may not be suitable for RLG due to its material properties in combination with the process conditions, although it leads to successful granulation in other wet granulation processes.

Higher tip speeds of the shaft were found to counteract the poor liquid distribution to a certain degree, but increased mechanical stress on the granules has to be considered. Additionally, it could be demonstrated, that the tip speed also influences the ring layer formation itself, which was shown to be crucial for sufficient granulation results. Since material properties and especially the changing interparticle cohesion by wetting the powder in the process are not considered by the Froude number, further studies should investigate the ring layer formation itself in more detail.

The similarities and differences in comparison with the literature on larger scale RLG and other continuous and batch wet granulation processes lay the foundation for deeper process understanding of the ring layer process, e. g. allowing for the correct application of already approved mechanistic models in future works. This knowledge on the small scale continuous RLG approach may stress its value for scale-up free application production of clinical samples as well as direct market supply for orphan drug products.

Future investigations should be conducted on the one hand to enhance the granulation yield and homogeneity by using more sensitive powder formulations as well as binder supply by spraying to overcome the differences with larger scale RLG processes. On the other hand, investigations should focus to generate a better mechanistic understanding of the ring layer process on the particle level.

## CRediT authorship contribution statement

**Lukas Bahlmann:** Writing – original draft, Visualization, Methodology, Investigation, Data curation, Conceptualization. **Jan Henrik Finke:** Writing – review & editing, Conceptualization. **Arno Kwade:** Writing – review & editing, Supervision.

## Funding sources

This work was supported by the German Research Foundation (DFG) [grant number 413141366].

## Declaration of competing interest

The authors declare the following financial interests/personal relationships which may be considered as potential competing interests: Lukas Bahlmann reports financial support was provided by German Research Foundation. Lukas Bahlmann reports article publishing charges was provided by Project DEAL. If there are other authors, they declare that they have no known competing financial interests or personal relationships that could have appeared to influence the work reported in this paper.

## Data Availability

Data will be made available on request.

## Appendix A Supplementary data

Supplementary material Image 1
