# Supplementary material for: Process characterisation of continuous ring layer wet granulation at small scale
Source: Int J Pharm X. 2025 Nov 23;10:100454. doi: 10.1016/j.ijpx.2025.100454 (PMC12702373; doi:10.1016/j.ijpx.2025.100454)
Supplement: Supplementary file 1 — Supplementary material [file mmc1.pdf]

Supplementary

S1) Granulator tool configuration

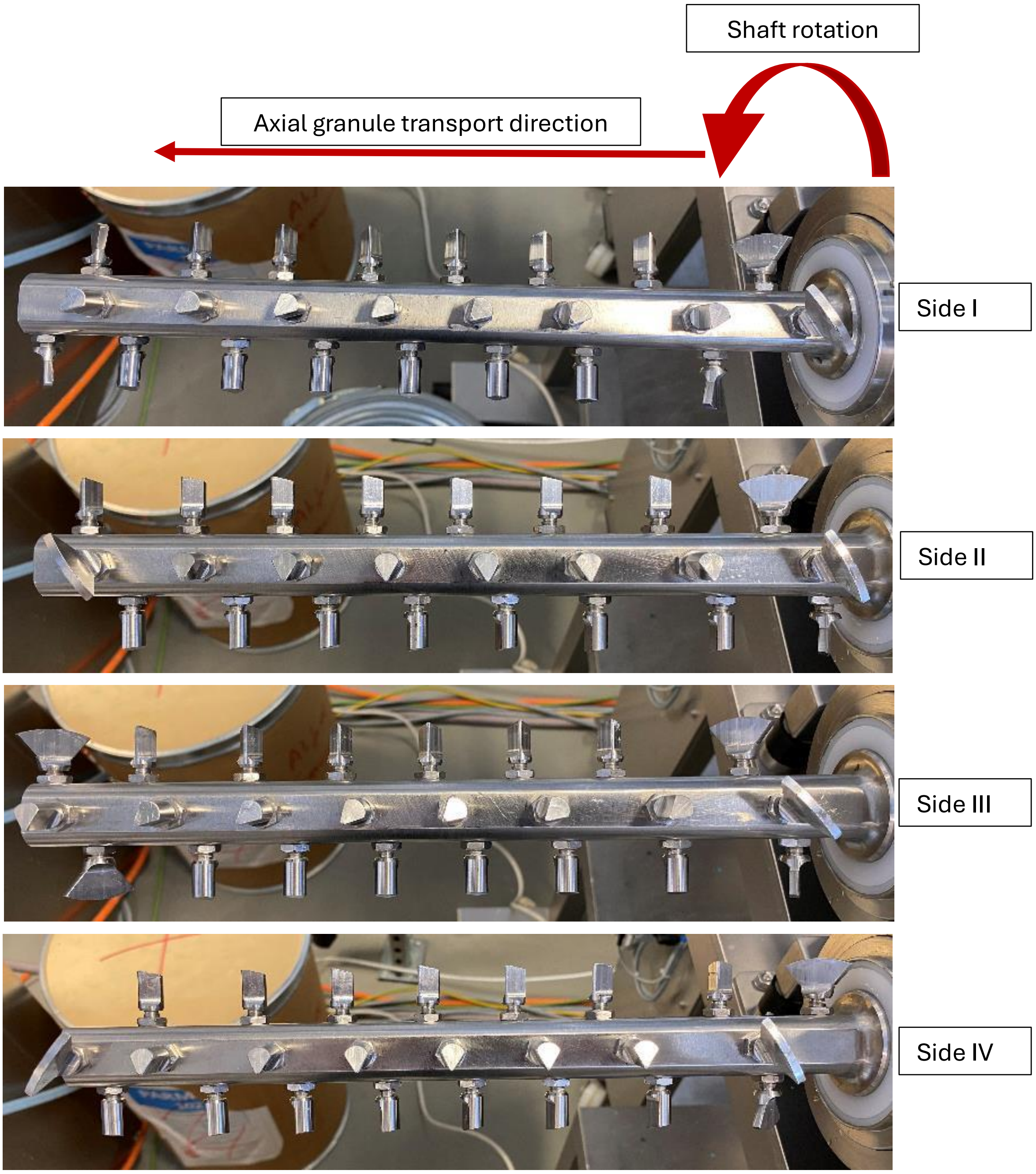

Fig. S1: *Tool configuration used in the present study as advised by the machine manufacturer.*

S2) Reproducibility

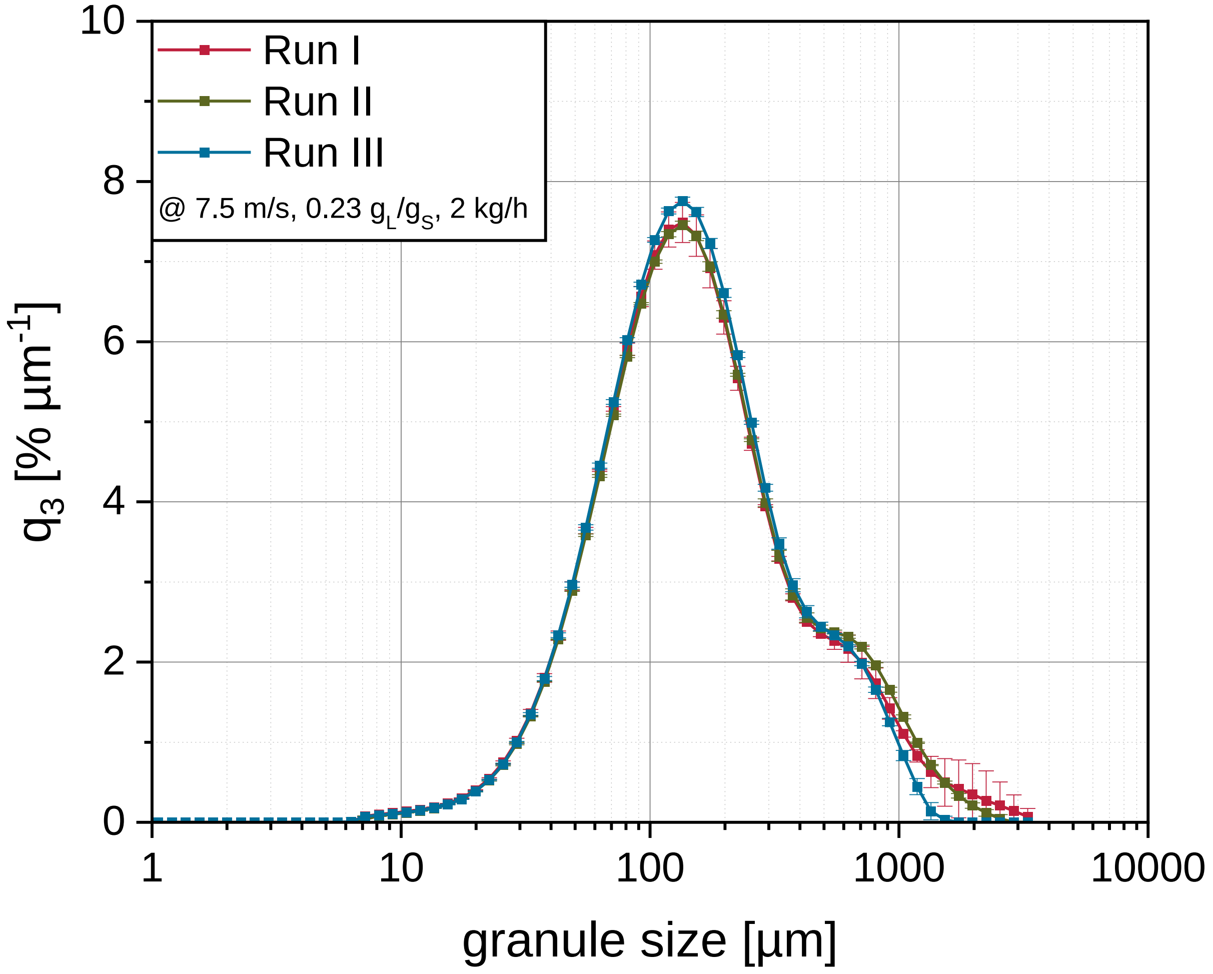

Fig. S2: *Granule size distributions of the three reproducibility experiments.*

Tab. S2: *Characteristic granule size values for the reproducibility runs.*

Run	$x_{10,3}$ [µm]	$x_{50,3}$ [µm]	$x_{90,3}$ [µm]
I	51.2 ± 0.7	145 ± 3	584 ± 51
II	51.8 ± 0.1	146 ± 0	597 ± 2
III	51.2 ±0.4	141 ± 1	486 ± 8

Reproducibility of the RLG process was tested by doing three independent granulation runs at a tip speed of 7.5 m/s, an L/S-ratio of 0.23 g_L/g_S and a powder feed rate of 2 kg/h. Shown above are the granule size distributions resulting from these three runs, which were obtained by laser diffraction measurements as described in chapter 2.3.1. A relative standard deviation of 1.74 % is obtained for the median granule sizes between batches, which is an indicator for sufficient reproducibility. Higher relative standard deviations are obtained at the larger percentiles of the distributions, which was also observed in studies on continuous twin screw granulation [1,2].

It has to be noted, that extensive studies on the process stability and the influence of different parameters on the variability/ reproducibility of the ring layer process are still missing and desireable, but would be beyond the scope of this work.

S3) Skewness of residence time distributions

The skewness s_θ^3 of the dimensionless RTDs $E(\theta)$ is calculated to determine the symmetry of the distributions:

$$\boldsymbol{s_\theta^3 = \int_0^\infty (\theta - \bar{\theta})^3 \cdot E(\theta) d\theta} \qquad (1)$$

With the the skewness, the shape of the RTD and thus the residence time behaviour can be described in more detail [3,4].

Tab. S3 *Skewness values of the RTDs under variation of pin tip speed, L/S-ratio and powder feed rate.*

Parameter variated	Value	Skewness s^3_θ [-]
Pin tip speed [m/s]	4.9	1.02
@ L/S = 0.130 g_L/g_S and	7.5	1.30
powder feed rate = 2 kg/h	9.9	0.68
L/S-ratio [g_L/g_S]	0.130	1.02
@ Pin tip speed = 4.9 m/s and	0.234	1.51
powder feed rate = 2 kg/h	0.420	1.50
Powder feed rate [kg/h]	2	1.02
@ L/S = 0.130 g_L/g_S and	4	1.04
pin tip speed = 4.9 m/s	6	0.49

S4) Hausner ratio

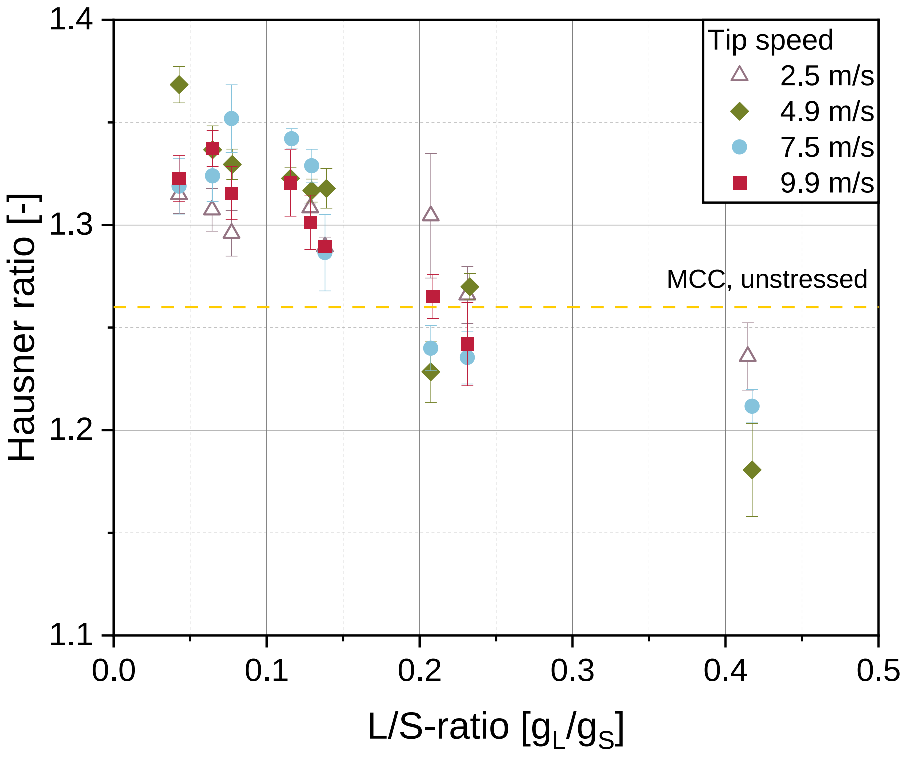

Fig. S4 *Hausner ratio determined with bulk and tapped density as a function of the L/S-ratio and shaft speed. Hausner ratio for the ungranulated MCC is given as a threshold value at 1.26.*

The Hausner ratio is used as measure of the dynamic compressibility and, by that, a rough estimator of the flow properties of the ring layer granules. Generally, with decreasing values for the Hausner ratio the flowability of the powder is improving [5]. For the determination, the unsieved granulated material was used to reflect the properties of the whole product. For higher L/S-ratios, smaller values for the hausner ratio were obtained. Thus, the flowability of the granulated powder is improved due to the higher share of larger, spherical granules. Interestingly, only from L/S-ratios from 0.234 g_L/g_S upwards, the flowability is superior compared to the flowability of the ungranulated MCC. On the one hand, this can be attributed to the already passable flowability of the used MCC grade with a Hausner ratio of about 1.26 [6,7]. On the other hand, this is caused by the relatively low share of actually granulated powder especially at small L/S-ratios, which leads to significant broadening of the particle size distribution. It is known that the broadening of the particle size distribution can lead to deteriorated flowability, even though mean particle size is larger which would be the case for the granulated powders [8].

References

[1] J. Vercruysse, U. Delaet, I. van Assche, P. Cappuyns, F. Arata, G. Caporicci, T. de Beer, J.P. Remon, C. Vervaet, Stability and repeatability of a continuous twin screw granulation and drying system, Eur. J. Pharm. Biopharm. 85 (2013) 1031–1038, http://doi.org/10.1016/j.ejpb.2013.05.002.

[2] E.I. Keleb, A. Vermeire, C. Vervaet, J.P. Remon, Twin screw granulation as a simple and efficient tool for continuous wet granulation, Int. J. Pharm. 273 (2004) 183–194, http://doi.org/10.1016/j.ijpharm.2004.01.001.

[3] D. Bošković, S. Loebbecke, Modelling of the residence time distribution in micromixers, Chem. Eng. J. 135 (2008) S138-S146, http://doi.org/10.1016/j.cej.2007.07.058.

[4] A.T. Harris, J.F. Davidson, R.B. Thorpe, Particle residence time distributions in circulating fluidised beds, Chem. Eng. Sci. 58 (2003) 2181–2202, http://doi.org/10.1016/S0009-2509(03)00082-4.

[5] H.H. Hausner, Friction conditions in a mass of metal powder, Int. J. Powder Metall. 3 (1967) 7–13.

[6] R.L. Carr, Evaluating flow properties of solids., Chem Eng. (1965) 163–168.

[7] E. Schlick-Hasper, J. Bethke, N. Vogler, T. Goedecke, Flow properties of powdery or granular filling substances of dangerous goods packagings—Comparison of the measurement of the angle of repose and the determination of the Hausner ratio, Packag. Technol. Sci. 35 (2022) 765–782, http://doi.org/10.1002/pts.2678.

[8] L.X. Liu, I. Marziano, A.C. Bentham, J.D. Litster, E.T. White, T. Howes, Effect of particle properties on the flowability of ibuprofen powders, Int. J. Pharm. 362 (2008) 109–117, http://doi.org/10.1016/j.ijpharm.2008.06.023.
